# Parasite Removal, but Not Herbivory, Deters Future Parasite Attachment on Tomato

**DOI:** 10.1371/journal.pone.0161076

**Published:** 2016-08-16

**Authors:** Muvari Connie Tjiurutue, Evan C. Palmer-Young, Lynn S. Adler

**Affiliations:** Biology Department, University of Massachusetts Amherst, 221 Morrill Science Center South, 611 North Pleasant Street, Amherst, MA, 01003, United States of America; Swedish University of Agricultural Sciences, SWEDEN

## Abstract

Plants face many antagonistic interactions that occur sequentially. Often, plants employ defense strategies in response to the initial damage that are highly specific and can affect interactions with subsequent antagonists. In addition to herbivores and pathogens, plants face attacks by parasitic plants, but we know little about how prior herbivory compared to prior parasite attachment affects subsequent host interactions. If host plants can respond adaptively to these different damage types, we predict that prior parasitism would have a greater deterrent effect on subsequent parasites than would prior herbivory. To test the effects of prior parasitism and prior herbivory on subsequent parasitic dodder (*Cuscuta* spp.) preference, we conducted two separate greenhouse studies with tomato hosts (*Solanum lycopersicum*). In the first experiment, we tested the effects of previous dodder attachment on subsequent dodder preference on tomato hosts using three treatments: control plants that had no previous dodder attachment; dodder-removed plants that had an initial dodder seedling attached, removed and left in the same pot to simulate parasite death; and dodder-continuous plants with an initial dodder seedling that remained attached. In the second experiment, we tested the effects of previous caterpillar damage (*Spodoptera exigua*) and mechanical damage on future dodder attachment on tomato hosts. Dodder attached most slowly to tomato hosts that had dodder plants previously attached and then removed, compared to control plants or plants with continuous dodder attachment. In contrast, herbivory did not affect subsequent dodder attachment rate. These results indicate that dodder preference depended on the identity and the outcome of the initial attack, suggesting that early-season interactions have the potential for profound impacts on subsequent community dynamics.

## Introduction

Throughout their life cycles, plants face many antagonistic interactions that often occur sequentially. Studies using herbivores have established two themes describing how prior antagonistic interactions affect subsequent interactions via changes in plant phenotypes: specificity of elicitation, in which a plant’s phenotype depends on the identity of the initial attacker [1, 2]; and specificity of effect, in which subsequent antagonists respond differently to a given plant phenotype [2, 3]. For example, damage to milkweed plants (*Asclepias syriaca*) by milkweed beetles (*Labidomera clivicollis*) increased latex production compared to controls, but damage by monarchs (*Danaus plexippus*) did not, indicating specificity of elicitation [1]. In the same study, beetles were not affected by previous damage from either conspecifics or monarchs, but monarchs were smaller on plants that had been damaged by either herbivore, indicating specificity of effect. In addition to inducing different secondary chemicals, plants may exhibit specificity of elicitation by releasing different volatile blends in response to damage by closely related herbivores [4]. Changes in volatile blends could then not only affect subsequent herbivore preference, but also attraction of natural enemies specific to each herbivore species [5].

Changes that occur in plant traits can depend on the identity of the plant as well as identity of the attacker [6–8]. Plant genotypes can vary in resistance, quality, or induced responses [9–11], and therefore can affect subsequent interactions differently. For example, the effects of previous feeding by meadow spittlebugs (*Philaenus spumarius*) on stem galler performance (*Eurosta solidaginis*) on goldenrod (*Solidago altissima*) could be positive, negative or neutral depending on plant genotype [12]. Genetic variation in *Oenothera biennis* plants explained 45–75% of the abundance of common herbivore species, and had cascading effects structuring higher trophic levels [13]. Genotypic variation among plants, and specificity of effect and elicitation can therefore have significant impacts on the composition and population density of herbivores.

Many previous studies have established the importance of specificity and genetic variation for the effects of prior herbivory on subsequent herbivores and pathogens [3, 7, 9, 14, 15]. However, plants also face attacks by other plants. Parasitic plants can severely impact growth and reproduction of their hosts [16], and play critical roles in structuring communities at multiple trophic levels [17, 18]. Some of the world’s most economically devastating agricultural pests are parasitic plants [19]. For example, dodder, *Cuscuta* spp., cause significant damage to a wide variety of agricultural crops including alfalfa, clover, potato, carrot, sugar beets, chickpea, onion, cranberry, blueberry, citrus, and tomato [20, 21]. Dodder forms close connections with its host using specialized organs called haustoria to uptake water and nutrients, and this intimate connection between the parasite and host makes dodder especially hard to control using conventional methods [22]. Despite the critical ecological and economic roles played by parasitic plants, little is known about how previous host interactions affect parasitic plant attachment.

Host quality and host defenses can influence parasite performance [16, 23]. For example, growth of the root hemiparasite *Melampyrum arvense* was much higher on a legume host than with two non-leguminous grass hosts [24]. Parasite performance can also be affected by host defenses [16, 25]. Some parasites may benefit from taking up host secondary metabolites due to reduced herbivory, indirectly increasing pollinator visitation and fruit set of the parasite [26]. Other studies report that higher host secondary metabolites may deter parasites [16]. Since parasitic plant performance depends on host quality, previous parasitism or herbivory could affect parasite preference. However, we know very little about how comparable the effects of prior parasite attachment and herbivory are in affecting subsequent interactions with parasites themselves.

A few studies have demonstrated the effects of host herbivory on subsequent interactions with parasitic plants. For example, host clipping early in the season reduced biomass of the hemiparasites *Rhinanthus minor* and *Pedicularis kansuensis* [27, 28]. In contrast, simulated grazing of two host species did not affect performance of the hemiparasite *Odontites litoralis* [29]. Although these studies provides a glimpse into the effects of simulated prior herbivory on subsequent plant-plant interactions, more studies involving real herbivory, other parasites and host plants are necessary to establish clear patterns.

We tested the effects of prior dodder (*Cuscuta* spp.) parasitism and herbivory on subsequent dodder preference using tomato host plants in separate greenhouse studies. We hypothesized that previous stimuli will result in specific adaptive changes, such that host plants with prior parasite attachment would become more resistant to parasites, and that prior parasite attachment should deter subsequent parasitism more than herbivory would. This study is unique in that, to our knowledge, no studies have examined the effects or prior parasitism on future attachment of parasitic plants.

## Methods and Materials

### Study system

Tomato (*Solanum lycopersicum*) is consumed worldwide and touted for its health benefits [30]. A wide range of tomato cultivars and hybrids are available commercially with varying chemical and nutritional characteristics, including high antioxidant content [30]. We used six different commercial Heinz hybrid cultivars (Heinz Seed Company, Tomato Hybrid seeds, Modesto, CA, USA) with varying known resistance to dodder. Heinz cultivars ‘9492’, ‘9553’, and ‘9992’ are considered relatively resistant to dodder parasitism due the parasite’s inability to form viable haustorial connections with the host stems [31], but resistance of tomato cultivars ‘3402’, ‘5608’ and ‘8504’ has not been assessed to our knowledge.

Dodder seeds were collected on 28 September 2008 from a commercial cranberry bog in Carver, MA (the owner gave permission to collect seeds on the farm). These seeds were used to carry out all experiments in this study. Identification of *Cuscuta* species can be challenging; polymerase chain reaction (PCR) based on DNA sections from dodder collected from several sites in this region indicated that plants were mostly *C*. *gronovii*, but with some *C*. *campestris* and possibly *C*. *compacta* co-occurring (K Ghantous, University of Massachusetts Cranberry Experiment Station, pers. comm.) [32]. Because we did not identify dodder to species level, we will refer to the parasite by genus name only.

### Plant propagation and herbivore rearing

We conducted two separate greenhouse experiments in consecutive years, 2014 and 2015. The first experiment, hereafter referred to as the *prior dodder experiment*, tested the effects of prior dodder parasitism on subsequent dodder preference across 6 tomato cultivars. The second experiment, hereafter referred to as the *prior herbivory experiment*, asked how prior herbivory and mechanical damage affected subsequent dodder preference using a single tomato cultivar.

Tomato seeds for the prior dodder experiment were planted using Black Gold seedling mix (Sun Gro Horticulture Distribution Inc., Agawam, MA USA) in 72-plug trays on 10 April 2014, and trays were placed in a mist house at ~ 18°C day and night. Plants were repotted into 10 cm pots using Black Gold potting mix (Sun Gro Horticulture Distribution Inc., Agawam, MA, USA) on 25 April 2014. Pots were placed in 10 cm saucers for bottom watering to prevent dislodging dodder seeds. Plants for the prior herbivory experiment were propagated in the same way the following year, and were started on 23 September 2015 and repotted on 8 October 2015.

Dodder seeds were scarified in batches of 100 (0.01 g) in a 2 mL microcentrifuge tube for approximately 3 minutes using a small dremel tool [33]. Seeds were then rinsed with tap water using a fine mesh strainer, placed in Petri dishes lined with 90 mm moistened filter paper, sealed with Parafilm (Bemis Company Inc., Oshkosh, WI, USA), and placed in an incubator at 23°C until seed germination approximately 2 days later.

Tobacco hornworm larvae (*Manduca sexta*) were obtained from a research laboratory (L. Schwartz, Department of Biology, University of Massachusetts, Amherst, MA; the colony was initiated with eggs obtained from APHIS, USDA, Otis, Buzzards Bay, MA, USA). Prior to the experiment, larvae were reared on an artificial diet [34] and maintained at 27°C at a 16h: 8h light-dark photoperiod.

### Experimental design and methods

#### Prior dodder experiment

We asked how prior dodder parasitism affected subsequent dodder attachment using three treatments. Control plants had no previous dodder attachment. Dodder-removed plants had an initial dodder seedling attached, removed by hand from the stem of the host, and left in the same pot to simulate parasite death after attachment. Some of the early intervention of controlling dodder involves dodder removal by farmers [35], and dodder could be left behind in bogs in this way. Dodder-continuous plants had an initial dodder seedling that remained attached and not disturbed. Following these treatments, a second dodder seedling was added to assess how initial interactions affected subsequent attachment as the response. With this design, we can compare (1) initial dodder attachment rate for all six tomato cultivars, using the initial dodder attachment as a measure of dodder preference, and (2) subsequent (second) dodder attachment due to both treatment and host cultivar.

The experiment used a randomized complete block design, with plants placed in blocks based on the same plant height as possible similarity in height. Plants were 3–4 weeks old. Each of the 30 experimental blocks consisted of 3 dodder treatments x 6 cultivars, for a total of 540 plants. Individual plants were placed at least 20 cm apart. The initial dodder seedling was placed 1 cm on the soil surface away from host stem in the dodder-continuous treatment and dodder-removed treatments. Although it was not the primary goal of our experiment, we monitored plants daily and recorded the date when this initial dodder attached (when dodder tightly coiled around the host stem with signs of haustorial swelling) or died as a measure of dodder preference across tomato cultivars. Two days after the first dodder attached, dodder was removed and left in the pot in the dodder-removed treatment, and left attached on the dodder-continuous plants. On the same day that the first dodder seedling was removed, another dodder seedling was added 1 cm away from the host stem to measure how dodder responds to previous attachment. The point of attachment for the second dodder seedling on the host was not controlled beyond seedling placement. Similarly, after two days of the first dodder attachment, a second dodder seedling was added to dodder-continuous treatments by placing dodder seedling on the soil surface 1 cm away from the host stem. Control plants received dodder seedlings on the same day that the dodder-continuous plants in their block received the second dodder. Plants were monitored daily, and the date when dodder attached or died was recorded as a measure of dodder preference. To analyze subsequent dodder attachment, we ultimately included only three cultivars from the original six because of low attachment by the initial dodder seedling on the three most resistant cultivars, resulting in low replication (fewer than 10 replicates per treatment for these cultivars). We measured plant height on the day we added the first dodder to all plants as a covariate in analyses.

#### Prior herbivory experiment

This experiment compared dodder attachment rate for mechanically damaged plants, caterpillar-damaged plants and control plants on one tomato cultivar, ‘H5608,’ chosen due to its high success of dodder attachment. Host plants were placed in 50 blocks based on plant height with one plant of each treatment (control, mechanical damage and caterpillar damage) per block for a total of 150 host plants. Similarly, individual plants were placed at least 20 cm apart from each other. Plants were 3–4 weeks old. To damage the host plants, we added one second instar tobacco hornworm larva to the caterpillar damage treatment using fine mesh bags (17.5 cm x 13 cm). We bagged 35 blocks on one day and the remaining 15 blocks on the next day due to limited bags. Plants in other treatments were bagged without larvae. Larvae fed on the plants for a day before removing bags. Any larvae that died were replaced and left for an additional day. Once the bags were removed, we used a pair of dissecting scissors in the mechanical damage treatment to simulate the same amount and distribution of herbivory as the caterpillar-damaged plant in that block. Bags were removed from control plants at the same time as other plants in that block. One day after removing the bags, we added a single dodder seedling 1 cm away from all host stems on the soil surface in that block. Plants were monitored daily, and the day when dodder attached or died was recorded as a measure of dodder preference. We measured host height and leaf length (mid-vein length of host’s longest leaf) on the same day we added dodder to use as covariates in analysis.

#### Statistical analysis

We used R version 3.2.1 for Mac [36] to carry out all statistical analyses. In both experiments, our response was the rate of dodder attachment, analyzed using a Cox proportional hazards mixed-effects model with maximum likelihood parameter estimation [37]. The rate of dodder attachment was analyzed as a survival object that included both whether the plant attached or died, and days until that event.

#### Prior dodder experiment

We tested for differences in initial (pre-treatment) rate of dodder attachment across all six cultivars as a measure of dodder preference using a Cox mixed-effects model. The model included cultivar as a fixed independent factor and block as a random factor. To analyze rate of attachment for the second, post-treatment dodder seedling, we ran a separate Cox mixed-effects model including treatment and the treatment by cultivar interaction term as additional fixed independent variables. For this second Cox model, we included only 3 cultivars due to low rates of dodder attachment by the initial dodder seedling in the other cultivars. Plant height was initially included as a covariate, but removed from both models because it did not explain significant variation in attachment rate (*P* > 0.62 for both). We conducted pairwise contrasts using the function glht in the package multcomp in R to test for differences in treatments and cultivars at α = 0.05 [38].

#### Prior herbivory experiment

As in the dodder experiment, we used a Cox mixed-effects model with rate of attachment as the response variable, treatment as a fixed independent factor, block as a random factor, and plant height and leaf length as covariates. Again, plant height did not explain significant variation and was excluded (*P* = 0.62), but leaf length was retained in the model.

## Results

### Prior dodder experiment

**Fig 1:**
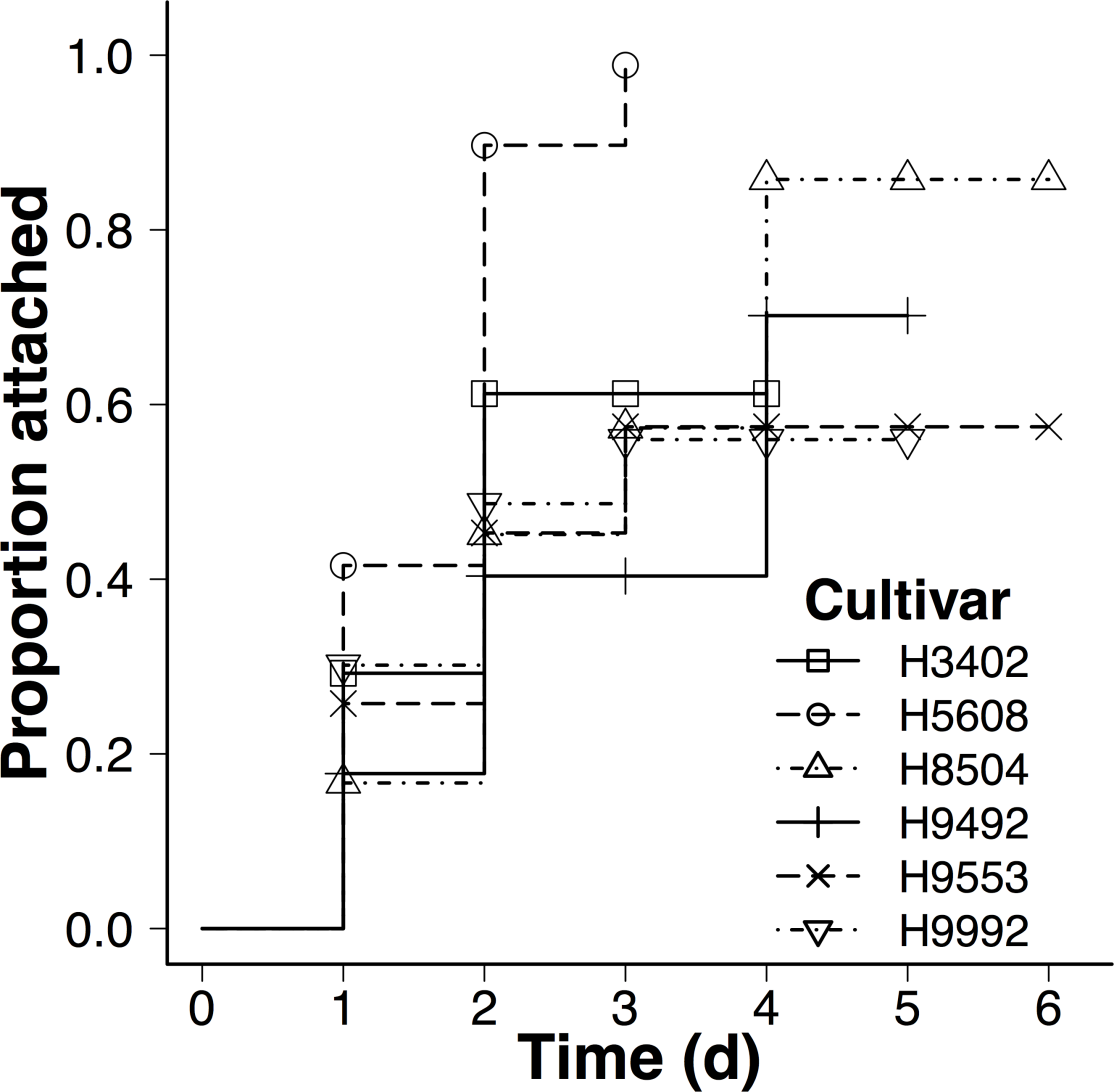
Differences in attachment rate of the initial dodder seedlings on 6 tomato cultivars. Lines shows proportion of dodder seedling attachment at each time point. Cultivar ‘H5608’ had significantly higher attachment rate compared to other cultivars (|*Z|* > 4.10, *P* < 0.001 for all).

Cultivar differences in initial dodder attachment. Attachment rates of the initial dodder seedling used for the manipulation differed with cultivar (Cultivar: χ^2^ = 65.85, df = 5, *P* < 0.0001). Dodder attached fastest to the susceptible cultivar H5608 compared to all other cultivars (|*Z|* > 4.10, *P* < 0.001 for all), but did not differ between any other cultivars (|*Z| <* 2.21, *P* > 0.23 for all; Fig 1).

**Fig 2:**
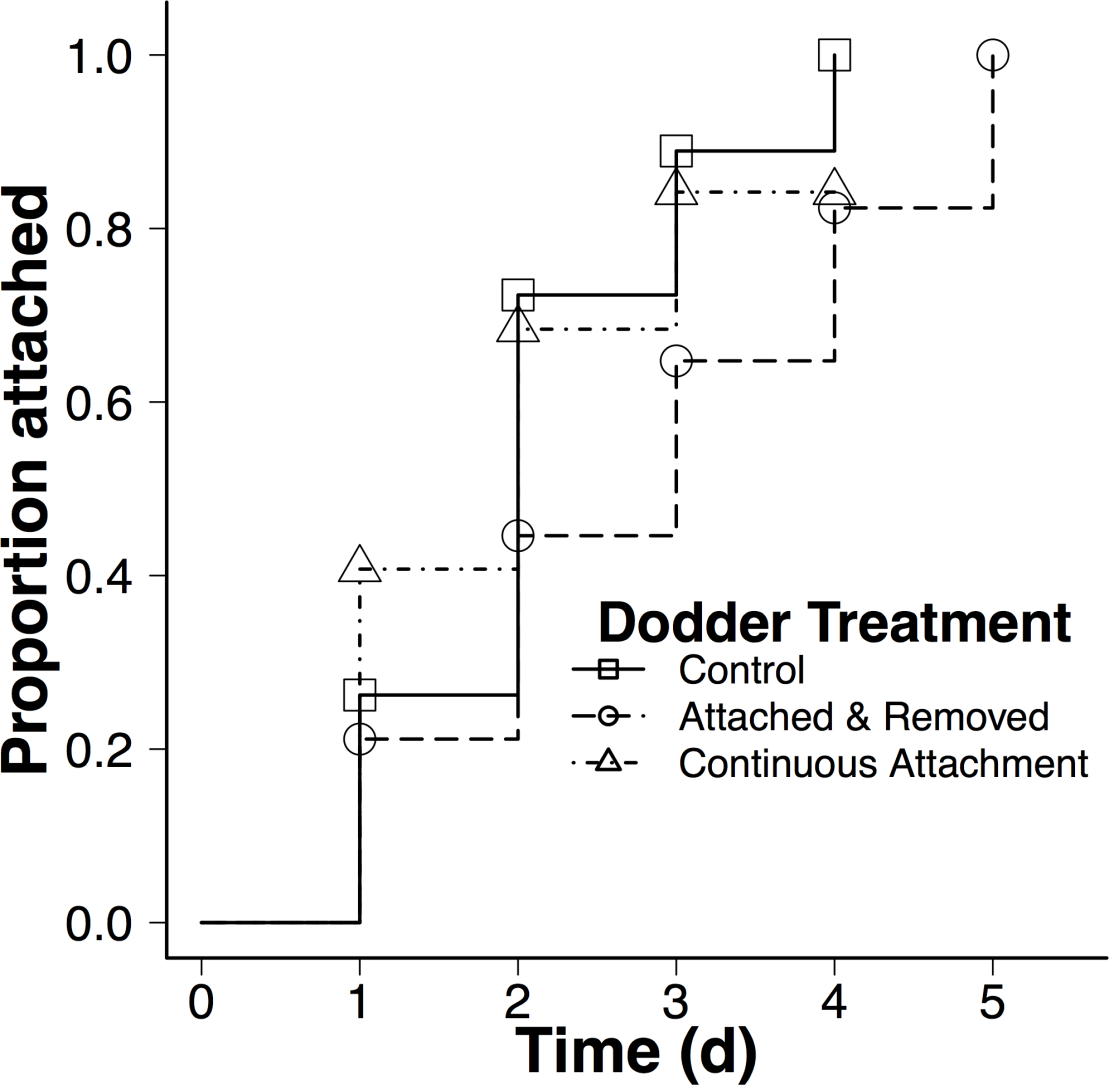
Effects of prior dodder treatment on the attachment rate of a second dodder parasite across 3 tomato cultivars. Lines indicate proportion of dodder seedling attachment at each time point. The ‘continuous attachment’ treatment line stops at day 4 because all dodder that did not attach by this point had died. Plants with the initial dodder removed had significantly lower attachment rate compared to control (Dodder-removed: hazard ratio vs control = 0.48, *Z* = -2.96, *P* = 0.0031) and plants with continuous dodder attachment (Dodder-continuous: hazard ratio vs control = 0.97, *Z* = -0.13, *P* = 0.900).

Effects of initial attachment on subsequent dodder attachment. Previous dodder parasitism affected subsequent dodder attachment (Treatment: χ^2^ = 10.08, df = 2, *P* = 0.0065). Compared to the control treatment, hosts with the initial dodder seedling attached and then removed experienced significantly slower attachment by the second dodder (Dodder-removed: hazard ratio vs control = 0.48, *Z* = -2.96, *P* = 0.0031). In contrast, dodder attachment did not differ in c the continuous dodder attachment treatment compared to the control treatment (Dodder-continuous: hazard ratio vs control = 0.97, *Z* = -0.13, *P* = 0.900; Fig 2). By day 3, only about 63% of dodder was attached to hosts in the dodder-removed treatment, compared to 90% of attached dodder to control hosts and 85% attached in the dodder-continuous treatment (Fig 2). Dodder attachment rate also varied across cultivars (Cultivar: χ^2^ = 11.23, df = 2, *P* < 0.0037), but there was no significant treatment by cultivar interaction (Treatment by Cultivar: χ^2^ = 0.98, df = 4, *P* = 0.91). Our conclusions were unaffected when we retained all 6 cultivars in our analysis, including those with low replication (see text and figures in S1 Supporting Information for detailed information).

### Prior herbivory experiment

**Fig 3:**
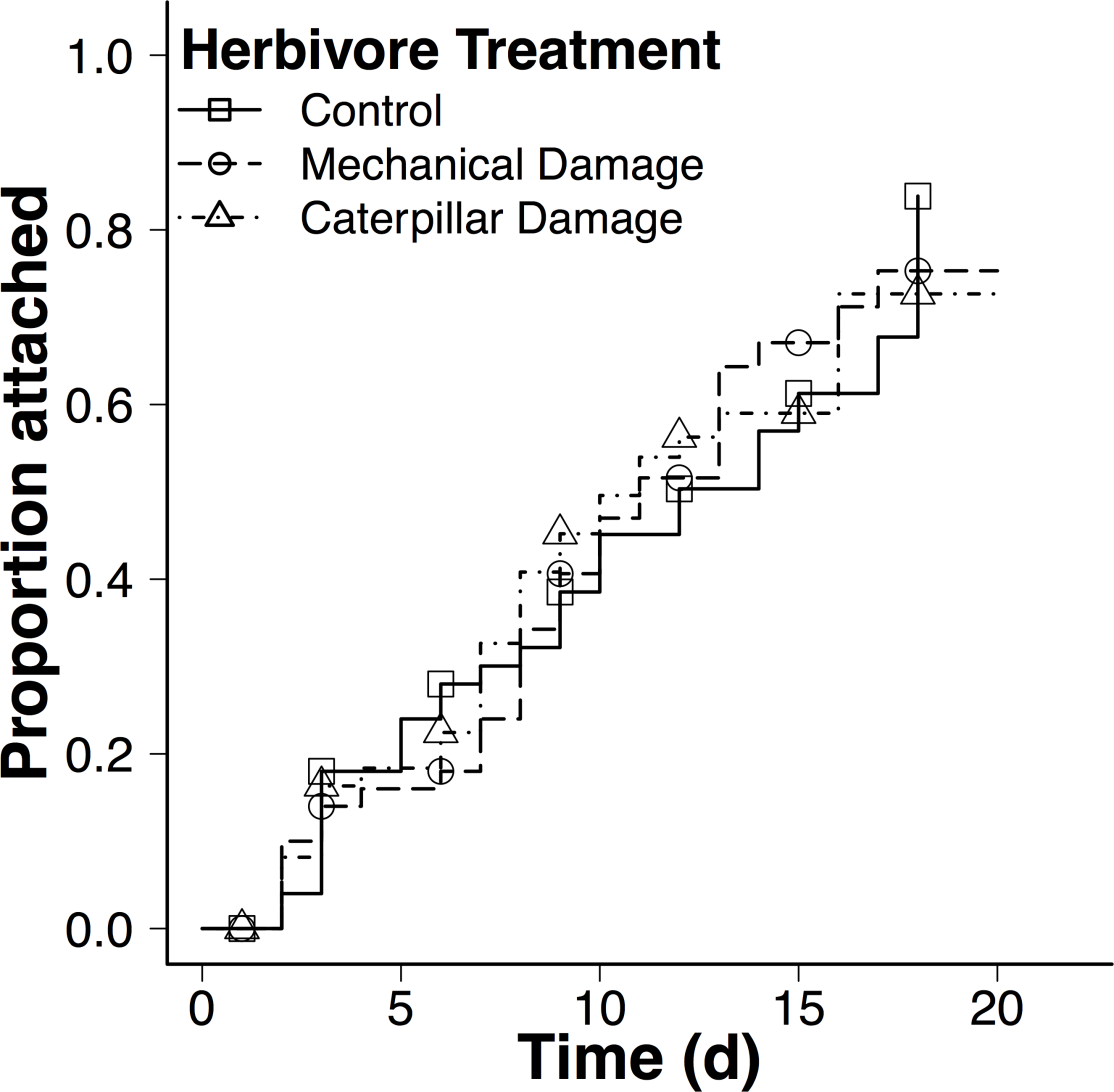
Effects of prior herbivory on the attachment rate of dodder plants on the ‘H5608’ tomato cultivar. Lines indicate proportion of dodder seedling attachment at each time point. There were no significant differences between treatments (Herbivory: hazard ratio vs. control = 1.10, *Z* = 0.36, *P* = 0.72; Mechanical: hazard ratio vs. control = 1. 14, *Z* = 0.51, *P* = 0.62).

There was no effect of herbivory or mechanical damage on dodder attachment (Treatment: χ^2^ = 0.28, df = 2, *P* < 0.87; Herbivory: hazard ratio vs. control = 1.10, *Z* = 0.36, *P* = 0.72; Mechanical: hazard ratio vs. control = 1. 14, *Z* = 0.51, *P* = 0.62; Fig 3). However, the covariate leaf length was marginally significant (Leaf length: χ^2^ = 2.91, df = 1, *P* = 0.087); dodder attached marginally slower to hosts with longer leaves (β = -0.22). We also note that the time to attachment and the proportion of dodder attachment was much longer and lower in the prior herbivory experiment compared to the prior dodder experiment, possibly due to colder temperatures or drier conditions, since the prior herbivory experiment was conducted in winter and the prior dodder experiment was conducted in late spring.

## Discussion

Dodder attached more slowly to hosts with previously attached dodder that were removed, compared to control hosts or hosts with continuous dodder attachment (Fig 2). We hypothesized that previous dodder attachment would induce host plant changes that affect subsequent dodder attachment, so this result was not entirely surprising. However, we also expected that any previous parasite attachment would deter subsequent parasitism, regardless of whether the parasite was then removed or remained attached. Although our experiments were not designed to assess mechanisms, it is possible that hosts with dodder removed released a different scent profile, repelling subsequent dodder plants compared to other treatments. Additionally, since we left the removed dodder seedling in the pot, the dying or dead dodder may have released a scent that deterred other dodder seedlings. Alternatively or in addition, failure to attach could be due to host defense mechanisms that operated after dodder encountered hosts but that prevented attachment.

Volatiles released from dodder itself could mediate attachment of subsequent dodder. Dodder (*C*. *pentagona*) can distinguish between preferred tomato host plants (*S*. *lycopersicum*) and non-preferred wheat host plants (*Triticum aestivum*) based on host scent [39]. If slow attachment was caused by repellent effects of the initial dying dodder seedling, then the effects of the dodder-removed treatment should still be observed even if the initial seedling did not attach. This hypothesis could be tested by comparing dodder responses to the effects of removing dodder completely after it attaches, versus removing dodder after it attaches but leaving the dying dodder in the pot. If the presence of dying dodder in the pot is sufficient to repel subsequent dodder attachment, then adding dead or decaying dodder to areas with high parasite infestations could provide environmentally friendly protection to valuable crops.

Induced host defenses could also mediate interactions between previous dodder and future attachment. In tomato, host defenses mediated by phytohormones can impact parasite performance [23, 25]. For example, *C*. *pentagona* grew larger on both jasmonic-insensitive and salicylic-deficient tomato hosts, suggesting that both jasmonic (JA) and salicylic (SA) responses may be effective against parasitic plants. Both JA and SA are involved in the hypersensitive response (HR) response, which is an effective defense against *C*. *reflexa* [31, 40], and JA-SA mutants generally lacked a noticeable HR [41]. Future research could investigate the role of host-induced defenses against dodder attachment using JA-SA insensitive mutant host plants. If slow attachment of dodder is due to induced defenses mediated by JA or SA, then we would expect no or reduced effect of initial dodder parasitism on subsequent attachment in JA- or SA-signaling mutant hosts.

The surprising effects of dodder removal on subsequent parasite attachment could be due to a complex interplay of signaling between hosts and parasites. Although this has not been demonstrated, we speculate that perhaps dodder can suppress or manipulate host defenses when attached to the host but not after removal, allowing plants to activate their defenses following unsuccessful attachment. Suppression of host defenses by herbivores is not uncommon [7, 8, 42, 43]. For example, feeding or application of oral secretions of *Manduca sexta* larvae to leaf punctures suppressed production of nicotine in *Nicotiana attenuata* plants compared to mechanically damaged plants [44]. In a more recent study, jasmonic acid accumulated in response to artificial damage but was suppressed by feeding of the pea aphid *Acyrthosiphon pisum* in broad beans, *Vicia faba* [45]. Future studies quantifying host JA and SA defenses in both dodder-continuous and dodder-removed treatments could provide some insights into the mechanisms mediating these interactions. Based on the results of our study, we predict that compared to dodder attachment and removal, continuous dodder attachment would suppress phytohormonal responses in hosts.

The different effects of the dodder-removed and dodder-continuous treatment on subsequent parasitism warrant further investigation to elucidate their ecological effects. The differences between these treatments suggest that the effects of prior interactions depend not just on the identity of the antagonist (i.e., herbivore vs. parasite), but also on the outcome of the interaction. Given that prior parasite attachment can reduce subsequent herbivory and deter pollinators [46], we suggest experiments testing whether these two dodder induction treatments vary in their effects on subsequent herbivore attraction, pollinator attraction, and fruit yield. Although tomato plants are largely self-fertile, pollinators are required to yield fruit [47], so the effects of parasites on pollinators are particularly relevant in agriculture. In addition, because dodder attachment can induce SA-based phytohormonal responses [48] normally associated with resistance to pathogens [6, 49], the effects of parasitic plants on susceptibility to bacterial and fungal tomato diseases such as fungal blights and bacterial canker could provide insight into the specificity of induction and variation according to the outcomes of previous parasitism.

Our results demonstrate that herbivory and prior dodder attachments had markedly different effects on subsequent dodder attachment. In contrast to prior dodder attachment and removal, prior mechanical damage and herbivory did not affect subsequent dodder preference (Fig 3). This was surprising, since many studies report that both caterpillar and mechanical damage can affect subsequent herbivore preference and performance [2], often with stronger effects of herbivory than mechanical damage due to oral secretions [44, 50]. We speculate that dodder may induce different plant defenses from herbivory in tomato. Plants can respond differently to specific herbivores, which influence subsequent interactions with the same host plant [1, 2, 14]. For example, two specialist herbivores preferred and performed better when fed leaves damaged by conspecifics than heterospecifics, and elicited qualitatively different secondary compound profiles across three *Solidago altissima* genotypes [9]. In another study, herbivory by two caterpillar species produced distinct volatile blends, and an egg parasitoid was attracted only to volatile blends produced by its host [51]. It is therefore possible that the dodder-removed treatment and tobacco hornworm damage elicited different defense responses in tomato that affect subsequent dodder attachments.

Potential differences in phytohormonal responses to parasitic plants and herbivores may provide an additional explanation for why herbivory did not affect subsequent parasitism. Dodder parasitism marginally induced SA production in cranberry hosts [52], and both SA and JA signaling pathways in tomato [48]. Generally, chewing insects such as caterpillars activate the JA-signaling pathways [6]. Since in our current study we used a chewing herbivore (*M*. *sexta*), we expect that herbivory should have induced JA production. Perhaps the activation of the SA signaling pathway is also required as a defense mechanism against dodder parasitism in tomato. It is also possible that the amount of damage due to herbivory or mechanical damage in our study was too low to influence dodder attachment. In many systems including tomato, increased damage levels induced higher levels of plant defense responses [53–55]. Although we did not measure amounts of damage in this study, future work could examine the influence of damage amount on dodder attachment given that induced defenses could vary with intensity of damage [56]. Collectively, our results show that prior herbivory and parasitism are important in shaping interactions between herbivores and parasitic plants with the same shared host.

In conclusion, we show that the effects of prior antagonistic interactions on dodder attachment depend not just on the identity of the previous antagonist, but also on the outcome of the previous interaction. Specifically, removal of dodder slowed subsequent dodder attachment, suggesting that removal of early dodder plants or adding dead dodder might provide agricultural avenues for controlling dodder. Cultivars also differed in their resistance, allowing for selection and use of the resistant cultivars in areas of high dodder infestations. Overall, our results are indicative that tomato responds differently to different antagonists, which could further shape interactions with other species in agro-ecosystems.

## Supporting Information

S1 Dataset Effects of prior dodder attachment on subsequent dodder attachment on 6 tomato cultivars. (XLS)

S2 Dataset Effects of prior herbivory on subsequent dodder attachment on 1 tomato cultivar (H5608). (XLS)

S1 Supporting Information Effects of prior dodder attachment on subsequent dodder attachment including all 6 tomato cultivars. (DOCX)
