# Supplementary material for: Parasite Removal, but Not Herbivory, Deters Future Parasite Attachment on Tomato
Source: PLoS One. 2016 Aug 16;11(8):e0161076. doi: 10.1371/journal.pone.0161076 (PMC4986975; doi:10.1371/journal.pone.0161076)
Supplement: S1 Supporting Information — (DOCX) [file pone.0161076.s003.docx]

# S1 Supporting information

**Article:** Parasite Removal, but Not Herbivory, Deters Future Parasite Attachment on Tomato

**Journal:** PLOS ONE

**Authors:** Tjiurutue MC, Palmer-Young EC, Adler LS.

**Correspondence:** Muvari Connie Tjiurutue, Biology Department, University of Massachusetts Amherst, 221 Morrill Science Center South, 611 North Pleasant Street, Amherst, MA 01003. Phone: 1-413-577-1824. Fax: 1-413-545-3243. Email: [connie.muv@gmail.com](mailto:connie.muv@gmail.com)

# S1 Supporting information

**Effects of prior dodder attachment on subsequent dodder attachment including all 6 tomato cultivars.**

Previous dodder parasitism affected subsequent dodder attachment when we retained all 6 cultivars in our analysis, including those with low replication (Treatment: χ^2^ = 10.93, df = 2, *P* = 0.0042; Cultivar: χ^2^ = 22.09, df = 5, *P* = 0.0005; Treatment by Cultivar: χ^2^ = 4.02, df = 10, *P* = 0.95;S1 Fig., S2 Fig.). Cultivars still attached more slowly to dodder-removed compared to control plants (Treatment: χ^2^ = 10.93, df = 2, *P* = 0.0042; Dodder-removed: hazard ratio vs. control = 0.58, *Z* = -2.77, *P* = 0.0057), but there was no difference in attachment between dodder-continuous and control treatments (Dodder-continuous: hazard ratio vs. control = 1.14, *Z* = 0.71, *P* = 0.48; S2 Fig.).

# Figure legends

**S1 Fig.** **Effects of prior dodder treatment on the attachment rate of a second dodder parasite across all 6 tomato cultivars, including 3 cultivars that were not retained for statistical analysis in the manuscript due to low sample sizes per treatment.** Lines indicate proportion of dodder seedlings attached at each time point. The ‘continuous attachment’ treatment line stops at day 4 because all dodder that did not attach by this point had died. When all 6 cultivars were included in analysis, plants with the initial dodder removed had significantly lower attachment rate compared to control and plants with continuous dodder attachment.

**S2 Fig.** **Differences in attachment rate of the second dodder seedlings on 6 tomato cultivars. Lines shows dodder seedlings remaining attached over time.** Cultivars ‘H9492 and H5608’ had significantly higher attachment rate compared to other cultivars.

**S2 Fig.**

**S2 Fig.**
